# Psychophysics with children: Investigating the effects of attentional lapses on threshold estimates

**DOI:** 10.3758/s13414-018-1510-2

**Published:** 2018-03-26

**Authors:** Catherine Manning, Pete R. Jones, Tessa M. Dekker, Elizabeth Pellicano

**Affiliations:** 1 Department of Experimental Psychology, University of Oxford, Anna Watts Building, Radcliffe Observatory Quarter, Woodstock Road, Oxford, OX2 6GG UK; 2 Centre for Research in Autism and Education (CRAE), UCL Institute of Education, University College London, London, UK; 3 UCL Institute of Ophthalmology, University College London, London, UK; 4 NIHR Moorfields Biomedical Research Centre, London, UK; 5 UCL Psychology and Language Science, University College London, London, UK; 6 Department of Educational Studies, Macquarie University, Sydney, NSW Australia

**Keywords:** Signal detection theory, Attention, Development

## Abstract

When assessing the perceptual abilities of children, researchers tend to use psychophysical techniques designed for use with adults. However, children’s poorer attentiveness might bias the threshold estimates obtained by these methods. Here, we obtained speed discrimination threshold estimates in 6- to 7-year-old children in UK Key Stage 1 (KS1), 7- to 9-year-old children in Key Stage 2 (KS2), and adults using three psychophysical procedures: QUEST, a 1-up 2-down Levitt staircase, and Method of Constant Stimuli (MCS). We estimated inattentiveness using responses to “easy” catch trials. As expected, children had higher threshold estimates and made more errors on catch trials than adults. Lower threshold estimates were obtained from psychometric functions fit to the data in the QUEST condition than the MCS and Levitt staircases, and the threshold estimates obtained when fitting a psychometric function to the QUEST data were also lower than when using the QUEST mode. This suggests that threshold estimates cannot be compared directly across methods. Differences between the procedures did not vary significantly with age group. Simulations indicated that inattentiveness biased threshold estimates particularly when threshold estimates were computed as the QUEST mode or the average of staircase reversals. In contrast, thresholds estimated by post-hoc psychometric function fitting were less biased by attentional lapses. Our results suggest that some psychophysical methods are more robust to attentiveness, which has important implications for assessing the perception of children and clinical groups.

## Introduction

A common way to characterize the sensitivity of a perceptual system is by measuring its perceptual threshold: the minimum stimulus intensity required to reach a specified level of performance (e.g., 70.7% correct; Green & Swets, 1974). Threshold estimates have been shown to improve during childhood for a range of visual tasks, including spatial contrast sensitivity (Bradley & Freeman, 1982; Ellemberg et al., 1999), temporal contrast sensitivity (Ellemberg et al., 1999) and motion perception (Hadad et al., 2011; Hayward et al., 2011; Manning et al., 2014). This protracted development is paralleled in other sensory modalities (e.g., audition: Fior, 1972; Jensen & Neff, 1993; Maxon & Hochberg, 1982; olfaction: Stevenson et al., 2007). Developmental scientists tend to assume that age-related reductions in psychophysical threshold estimates reflect real differences in sensitivity. Yet, it is possible that higher-level factors, such as inattentiveness, reduced motivation, or response bias, contribute to age-related changes in threshold estimates (Wightman & Allen, 1992). Thus, to fully understand the development of perceptual systems, it is important to investigate the extent to which these higher-level factors may impact on children’s psychophysical threshold estimates.

Here, we operationalized inattentiveness in terms of attentional lapses: responses made independent of the stimulus level (Kingdom & Prins, 2010; Madigan & Williams, 1987). Attentional capacities go through considerable developmental changes throughout childhood (Rueda et al., 2004) and, accordingly, lapses are often elevated in children compared to adults (Halliday et al., 2008; Manning et al., 2014; McArthur & Hogben, 2012; Talcott et al., 2002; see also Nardini et al., 2010). Simulations suggest that attentional lapses contribute to elevated and more variable threshold estimates in child populations (Roach et al., 2004; Wightman et al., 1989; Wightman & Allen, 1992; Witton et al., 2017).

It is unclear how the deleterious effect of inattentiveness may vary depending on the psychophysical procedure used. Answering this question is important for ensuring the robustness of findings in populations with limited attention such as children, and also elderly and clinical groups. Typically, when estimating thresholds in children, researchers and clinicians use psychophysical procedures that have been designed almost exclusively for use with adults. These include both non-adaptive and adaptive methods. Non-adaptive methods, such as the Method of Constant Stimuli (MCS), test responses at predefined stimulus levels, whereas adaptive methods (e.g., transformed staircases: Levitt, 1971; QUEST: Watson & Pelli, 1983; PEST: Taylor & Creelman, 1967) use previous responses to guide which stimulus levels are presented next, in order to “home in” on the threshold. Previous research suggests that different procedures converge on comparable estimates of thresholds in adult participants (Amitay et al., 2006; Leek et al., 2000; Madigan & Williams, 1987; Shelton & Scarrow, 1984; Shelton et al., 1982; but see also Kollmeier et al., 1988), but this finding may not generalize to observers with elevated lapse rates, such as children.

Adaptive methods have intuitive appeal when testing children. In these methods, trials are placed at stimulus levels strategically chosen to be informative of the observer’s threshold (Kingdom & Prins, 2010; Leek, 2001). While approaches differ, all adaptive methods are designed to estimate thresholds in fewer trials than non-adaptive methods (Macmillan & Creelman, 1991). Fewer trials are generally preferable when testing children, as they have the potential to minimize fatigue and/or boredom effects. Furthermore, unlike non-adaptive methods, stimulus intensities do not need to be predefined before the testing session, which is useful when there is high uncertainty about the observer’s threshold (King-Smith et al., 1994), as in the case of children whose thresholds may differ widely from those of their peers. Instead, other choices have to be made, including the selection of appropriate starting points, priors, and step-sizes (García-Pérez, 2011; Kaernbach, 1991; Watson & Pelli, 1983; Wetherill & Levitt, 1965).

While adaptive procedures have clear advantages compared to non-adaptive procedures for testing participants who cannot tolerate large numbers of trials, they may suffer from an important drawback. Because the next stimulus is selected based on previous trial performance, a lapse may cause the adaptive procedure to deviate away from the most efficient presentation sequence, potentially leading to unreliable estimates of threshold, especially when they occur early on in the testing session (Gu & Green, 1994; Kingdom & Prins, 2010). Threshold estimation techniques that fit the whole psychometric function, such as MCS and “hybrid” versions of adaptive methods (Hall, 1981) may minimize this effect of attentional lapses, as the lapse rate can be estimated from the psychometric function and accounted for (Dakin & Frith, 2005; Prins, 2012; Wichmann & Hill, 2001).

Buss et al. (2001) previously compared the use of different psychophysical procedures in children. They investigated auditory detection threshold estimates in a three-alternative-forced-choice task in 6- to 11-year-olds (n = 23) and adults (n = 13), comparing three procedures: a 1-up 3-down Levitt staircase (Levitt, 1971), a maximum likelihood estimation procedure (MLE; Green 1993), and MCS. Participants were first presented with the staircase and MLE procedures, and resulting threshold estimates were then used to select MCS stimulus intensity levels. Buss et al. reported that the three procedures yielded comparable threshold estimates, thus concluding that the choice of psychophysical procedure is not of great importance when assessing children’s auditory detection thresholds. The aim of the current study was to expand on this work by directly testing the effects of attentional lapses on children’s threshold estimates, using a large dataset combined with computer simulations.

We asked children aged 6–9 years and adults to discriminate between two sequentially presented random-dot stimuli moving at different speeds in a two-alternative forced-choice paradigm. We used three psychophysical procedures: QUEST (Watson & Pelli, 1983), a 1-up 2-down staircase, and MCS, to obtain discrimination threshold estimates, defined as the stimulus level leading to a 70.7% chance of a correct judgment about which stimulus moved faster. Unlike in Buss et al.’s study, we tested the more typical scenario where MCS stimulus levels are not selected using preceding staircase procedures. We chose a speed discrimination task for two reasons. First, speed discrimination threshold estimates have been shown to reduce with age, following a relatively protracted rate of development that only reaches adult-like levels by mid-to-late childhood (Manning et al., 2012). Second, young children show considerable between-participants variability in their speed discrimination threshold estimates, particularly for slow speeds (Ahmed et al., 2005; Manning et al., 2012). Increased attentional lapses could contribute to both elevated speed discrimination threshold estimates and between-participants variability in young children. We obtained a coarse measure of attentiveness from the number of incorrect responses to randomly-placed “easy” catch trials (Treutwein, 1995), where a random response at 10% of trials (0.1 lapse rate) would yield an expected error rate of 5% on catch trials in the 2AFC task. In addition to these empirical data, we used Monte Carlo simulations to assess whether attentional lapses could explain the pattern of threshold estimates across methods obtained in our participants.

We had three main research questions. First, we tested whether children have more attentional lapses than adults using errors in catch trials as a proxy. We predicted that they would, based on previous research (Manning et al., 2014; Talcott et al., 2002). Second, we investigated whether speed discrimination threshold estimates varied across procedures, and whether the effect of procedure interacted with age group. Third, we assessed the effect of inattentiveness on different threshold estimation techniques using simulations to investigate whether any differences between threshold estimates across conditions in our participants could be explained by a differential effect of attentional lapses.

## Methods

### Participants

Three groups of participants were tested: 31 children in Key Stage 1^1^ (KS1) (M = 6.80 years, range 6.19–7.40 years, 18 females); 39 children in Key Stage 2 (KS2) (M = 8.39 years, range 7.53–9.27 years, 21 females), and 19 adults (M = 25.12 years, range 20.61–33.41 years, 10 females). Children were recruited from schools in the Greater London area, and adult participants were recruited through the UCL Institute of Education and community contacts. Parents completed a brief questionnaire about their child’s vision, and normal or corrected-to-normal visual acuity was confirmed by binocular testing with the Cambridge Crowding cards for children, and with a Snellen acuity chart for adults. Normal acuity was defined as a binocular crowded-letter acuity of 6/9 or better for children aged 6–8 years.

Analyses of catch trials were conducted with the full cohort, but threshold estimates could not be reliably computed for all participants in all conditions (see Data Screening and Analysis). The analysis on threshold estimates therefore involved a subset of 26 KS1 children (17 female), 32 KS2 children (17 female) and 12 adults (seven female).

### Apparatus and stimuli

**Fig. 1:**
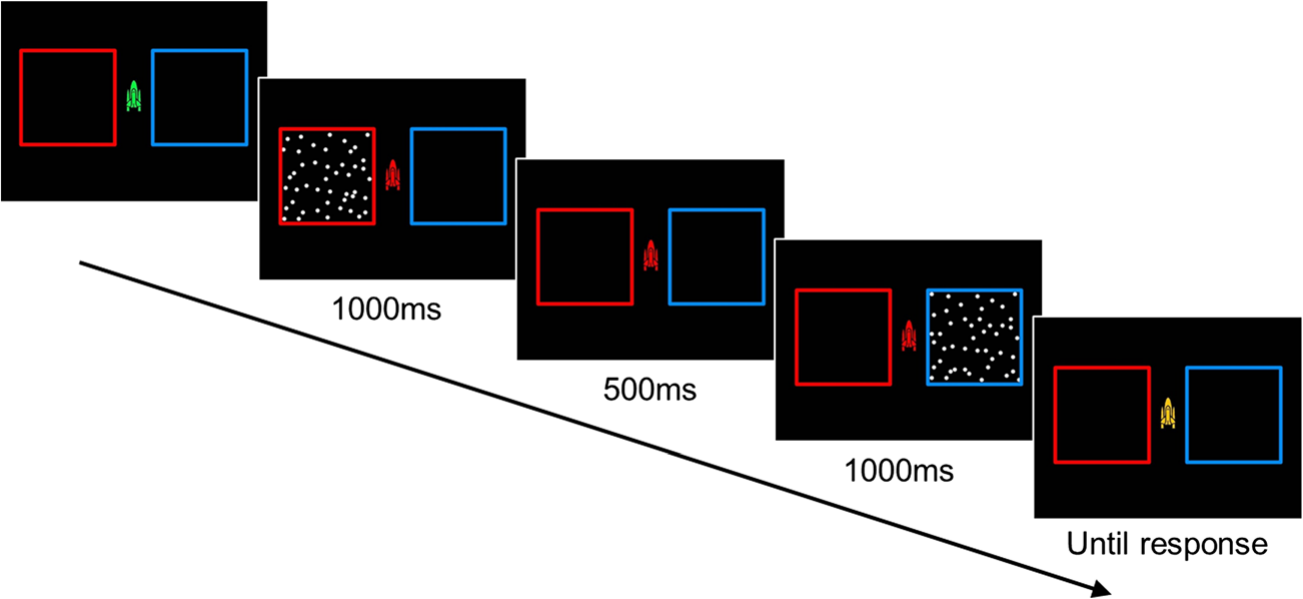
Representation of trial sequence. Reference and comparison stimuli were separated spatially and temporally. The order of presentation (reference or comparison first) and spatial location were randomized on each trial

The stimuli were presented using MATLAB (The Mathworks Ltd.) using the Psychophysics Toolbox (Brainard, 1997; Kleiner, Brainard, & Pelli, 2007; Pelli, 1997). The screen was black with a central fixation point (1.54° × 3.12°) in the shape of a rocket that changed color to mark trial events (Manning et al., 2012). There was a red square border (11° × 11°) to the left and a blue square border (11° × 11°) to the right of fixation. The stimuli were white random dot patterns moving with 100% motion coherence within either border (see Fig. 1). In each stimulus, there were 100 white dots of 0.34° diameter and a limited lifetime of five monitor refreshes (~83 ms).

### Procedure

The speed discrimination task was based on Manning et al. (2012). In each trial, a reference and a comparison stimulus were presented sequentially for 1,000 ms, separated by an interstimulus interval of 500 ms. A stimulus in the left (red) border was followed by a stimulus in the right (blue) border, and vice versa (see Fig. 1). The order of presentation of the reference and comparison stimulus (first or second interval) was randomized on each trial. The reference stimulus moved at 1.5 °/s, and the speed of the comparison stimulus varied above the reference speed.

The task was presented in the context of a space-themed “game,” to enhance children’s motivation and attention throughout the task. Participants were asked to determine whether the red or blue rocket had “stars” travelling faster past the window. Participants were presented with an initial demonstration and criterion phase, followed by practice and threshold estimation phases for each procedure. The initial phases served to cement the observer’s strategy before obtaining thresholds.

### Demonstration and criterion phase (“Space game warm-up”)

The experimenter explained the task to participants using a minimum of four demonstration trials. Participants indicated which set of “stars” moved faster. The experimenter replayed these initial demonstration trials if the child did not appear to understand this part of the procedure. Next, children were presented with up to 20 criterion trials that had a comparison speed of 8 °/s. Visual and verbal feedback about performance were provided. Children were required to reach a criterion of four consecutive correct responses in order to proceed to the next phase, to ensure task understanding.

### Practice phase (“Practice round”)

The practice phase consisted of eight trials with eight comparison speeds presented in a fixed order: 4.5, 3.75, 3, 2.625, 2.25, 1.875, 1.8, and 1.65 °/s. Participants received visual and verbal feedback about performance as before, but were not required to reach a criterion for correct responses.

### Threshold estimation phase (“Competition round”)

The comparison speed in the threshold estimation phase was guided by one of three procedures: QUEST (Watson & Pelli, 1983), 1-up 2-down staircase (Levitt, 1971), or MCS. Further details of each of these procedures are provided below. To allow comparability between conditions, all procedures were terminated after a fixed number of trials. Sixteen catch trials with a comparison speed of 8 °/s were included in each procedure to estimate attentional lapses (Treutwein, 1995). No veridical feedback was given regarding performance in the threshold estimation phase, although general encouragement was given throughout*.* The trials were separated into four equally sized blocks, and after each block, the participant was presented with a score and the score of an opponent. These scores were designed to aid motivation but did not reflect actual performance (see Manning et al., 2014).

### QUEST

The QUEST procedure was similar to that described by Manning et al. (2012), and was implemented using the QUEST toolbox in Psychtoolbox (Watson & Pelli, 1983). There were two tracks of 32 trials that varied in whether the reference stimulus or comparison stimulus was presented first. Tracks were interleaved to reduce trial-to-trial dependencies. An additional 16 catch trials were presented at random trial positions yielding 80 trials in total. The initial comparison speed (i.e., prior expectation) for each QUEST was derived from the estimated thresholds for each age group in Manning et al. (2012): 4.24 °/s for 6- to 7-year-olds, 3.80 °/s for 8- to 9-year-olds and 2.26 °/s for adults, with a standard deviation of 20. This prior was intentionally wide, to ensure that the prior did not exert a strong influence on the values tested (see Alcalá-Quintana & García-Pérez, 2004; Kingdom & Prins, 2010). Each QUEST had a beta value of 3.5 (corresponding to the slope of the expected Weibull function), and a lapse rate set to 0.02. The quantile method was used to recommend the next testing value (Pelli, 1987) using linearly spaced stimulus unit values (°/s). Jitter was added to the suggested values, to avoid participants becoming frustrated or demotivated if too many trials were placed near threshold (Watson and Pelli, 1983). The inclusion of jitter also ensured that a range of testing levels were presented, facilitating the fitting of psychometric functions. Jitter values were randomly selected from a normal distribution centered on zero with a standard deviation of 0.5. The suggested stimulus values were only accepted if they fell between 1.5 °/s and 8 °/s to ensure that the speed of catch trials was not exceeded. When QUEST suggested a value outside of this range, a randomly selected value from the acceptable range was chosen. During analysis, the threshold was evaluated at the 70.7% threshold level, to allow comparison with the staircase.

### Staircase

As in the QUEST procedure, the staircase procedure consisted of two interleaved tracks of 32 trials, with an additional 16 catch trials. The tracks were 1-up 2-down Levitt staircases, estimating 70.7% correct (Wetherill & Levitt, 1965). The staircases started at the same point as the QUEST functions. Initially, an incorrect response would increase the comparison speed by 0.2 times, and two consecutive correct responses would reduce the comparison speed by 0.2 times. The step size was reduced to 0.1 and 0.05 times the stimulus value after the first and second reversals, respectively. The larger initial step size was designed to minimize any effect of an inappropriate choice of starting point. The staircase values were subject to an upper limit of 8 °/s and a lower limit of 1.5 °/s as in the QUEST procedure.

### Method of Constant Stimuli (MCS)

The MCS procedure presented 16 trials at each of five levels of comparison speed in a random order. The comparison speeds were selected to span the expected threshold (Kingdom & Prins, 2010) for each age group (Manning et al., 2012). In the KS1 children, the comparison speeds were 2.19, 2.87, 4.24, 5.61, and 6.99 °/s; in the KS2 children, the comparison speeds were 2.08, 2.65, 3.80, 4.95, and 6.10 °/s; and in the adults the comparison speeds were 1.69, 1.88, 2.26, 2.64, and 3.02 °/s. As in the adaptive procedures, 16 catch trials were included with a comparison speed of 8 °/s for all participants, yielding a total of 96 trials for this procedure. The temporal order (reference stimulus vs. comparison stimulus presented first) was randomized on each trial.

### General procedure

The procedure was approved by the Institute of Education’s Faculty Research Ethics Committee. All adult participants and parents of child participants gave informed consent, and children provided verbal assent. Child participants were seen individually at school and adult participants were seen at the Institute of Education or another convenient location. Procedures were presented in separate sessions to minimize fatigue, with the first and last sessions spanning no more than 9 days, and each session lasted approximately 15 min. The order of presentation of the different procedures was counterbalanced across participants. In the first session, participants completed the demonstration and criterion phase, followed by the practice and threshold estimation phase for one procedure. In the second and third sessions, participants were reminded of the task before proceeding to the practice and threshold estimation phases for the second and third procedures, respectively.

Participants were tested binocularly in a dimly illuminated room at a viewing distance of 50 cm from the computer screen, fixed using a chin-rest. Participants were instructed to maintain central fixation throughout stimulus presentation, and received regular reminders to do so. Child participants gave responses verbally or by pointing, and the experimenter pressed the corresponding response key. The experimenter continuously monitored children’s eye movements and initiated trials only when the child was fixating appropriately. Adult participants made their responses by directly pressing the response keys.

### Data analysis

As an index of attentiveness, we computed the proportion of catch trials for which participants gave an incorrect response. We used non-parametric analyses when analyzing group and condition differences in catch trial error rates, as the data were highly skewed, with the majority of error rates being zero and unamenable to transformation. These analyses were conducted with the full sample of 31 KS1 children, 39 KS2 children, and 19 adults.

**Fig. 2:**
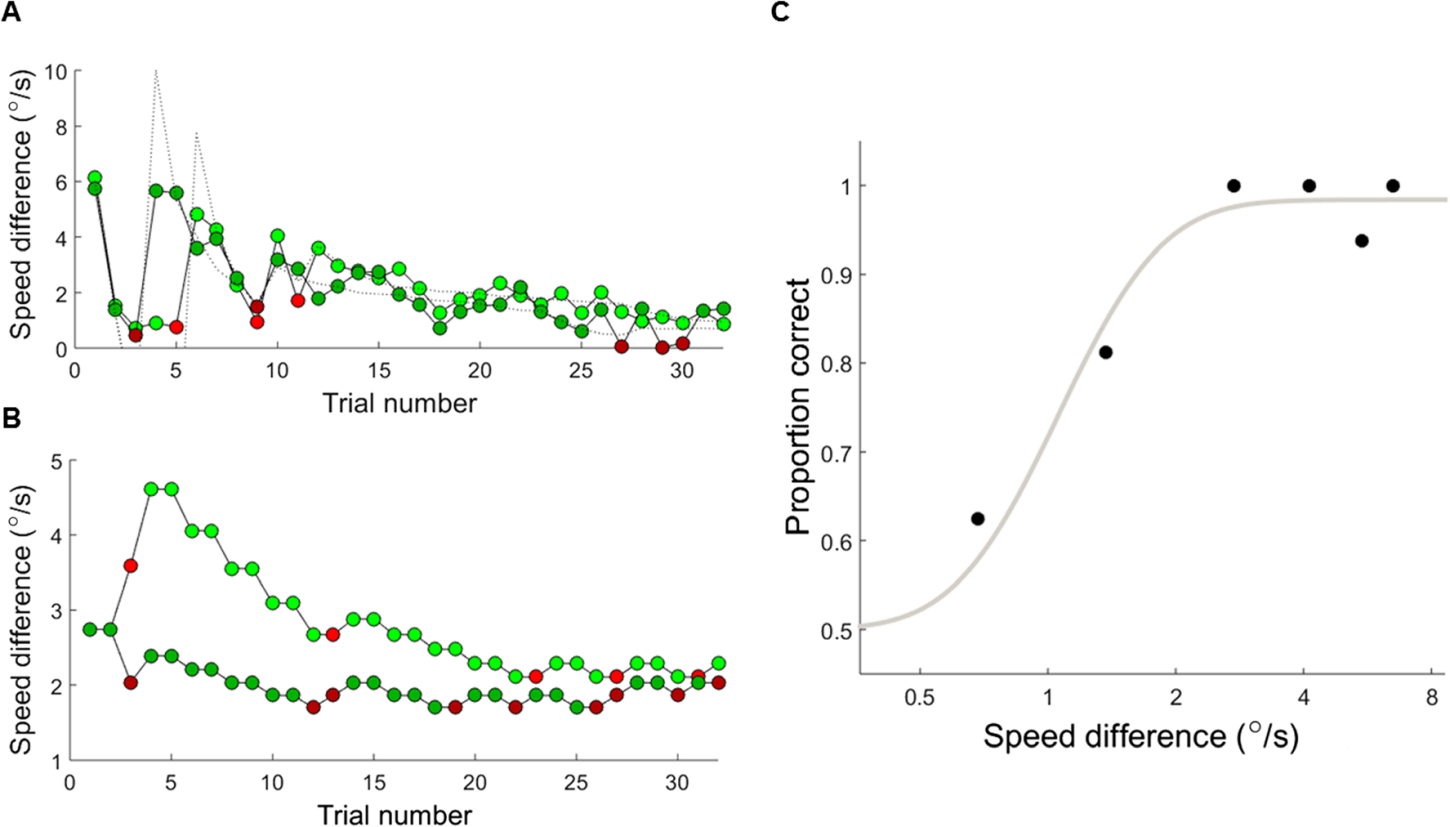
Example datasets for each psychophysical procedure. (**A**) Example dataset for the QUEST condition, belonging to a participant in the KS1 group. The speed difference is the difference between the comparison speed and the reference speed (1.5 °/s). The values suggested by the QUEST functions are shown as black dotted lines, and the presented values (after trimming within the allowable stimulus range and adding jitter) are shown as filled markers. Green markers indicate correct responses and red markers indicate incorrect responses. (**B**) Example dataset for the staircase condition, belonging to a participant in the KS1 group. As above, green markers indicate correct responses and red markers indicate incorrect responses. (**C**) Psychometric function fit to an example dataset for the MCS condition, in which the proportion of correct responses is plotted for each speed difference between the comparison and reference stimulus

To estimate thresholds, we first fit psychometric functions to the raw data from each individual participant in each procedure (including the adaptive tracks, i.e., the “hybrid adaptive procedure,” Hall, 1981; Kingdom & Prins, 2010). The catch trials were included in the fit and we did not bin trials in the adaptive procedure. We fitted bootstrapped cumulative Gaussian functions with 200 runs, using the psignifit toolbox (see http://bootstrap-software.org/psignifit/), a software package that implements the maximum-likelihood method described by Wichmann and Hill (2001). The proportion of correct responses was plotted as a function of the difference in speed between the reference and comparison stimuli on a log scale (Fig. 2), with the guessing rate (gamma) fixed at 0.5. Lapse rate (lambda) was a free parameter allowed to vary between 0 and 0.1 with equal probability of values across this range. The threshold was defined as the speed difference at which 70.7% of responses were correct. Participants were excluded from the threshold analysis if negative slopes were obtained (QUEST: n = 1; staircase: n = 2) or if the threshold estimates fell outside of the tested stimulus range (QUEST: n = 8; staircase: n = 9; MCS: n = 2). It is possible that the QUEST and staircase had higher exclusion rates than MCS because MCS included more trials. Notably, many adult participants and some child participants had threshold estimates below the minimum presented stimulus, which seemed to be caused by too few trials targeting below-threshold. Although these data indicate good performance, these threshold estimates are unreliable as they involve extrapolating beyond the tested values. We excluded these participants from the threshold analysis to ensure that we could compare estimates reliably across methods, resulting in a smaller dataset of 26 KS1 children, 32 KS2 children, and 12 adults for these analyses.

Additionally, we computed threshold estimates specific to the adaptive track used. For each participant’s data in the staircase condition, we averaged the last even number of reversals, omitting the first two reversals, for each staircase, and averaged these across the two runs to get a single threshold estimate. For one KS1 child, too few reversals were obtained to compute a threshold estimate, so this participant was excluded from analysis involving the staircase reversals. For the QUEST condition, we obtained the mode of the posterior probability density function (Watson & Pelli, 1983) for each QUEST track using the QUEST toolbox in Psychtoolbox and averaged these within participants to get a single threshold estimate.^2^

### Simulations

To model the effects of attentional lapses on threshold estimates, we ran a set of 2,000 Monte Carlo simulations for each psychophysical procedure, using a simulated observer with progressively greater lapse rates. Trial values were presented to the simulated observer in the same way as the youngest, KS1 children. The simulated observer responded on the basis of a cumulative log-Gaussian psychometric function with a standard deviation (internal noise) of .20 and a mean of .02 in log units. This function was based on the MCS data from the KS1 observer shown in Fig. 2C with a perceptual threshold of .97 °/s. The lapse rate corresponded to the proportion of trials on which the simulated observer would respond randomly (see also Gu & Green, 1994; Madigan & Williams, 1987; Prins, 2012). Thresholds were estimated in the same way as those for the real datasets, and again, we filtered out threshold estimates falling outside of the stimulus range and psychometric functions with negative slopes. The code and resulting simulations can be found at: https://osf.io/ne2c8/.

## Results

### Characterizing the attentiveness of children

**Fig. 3:**
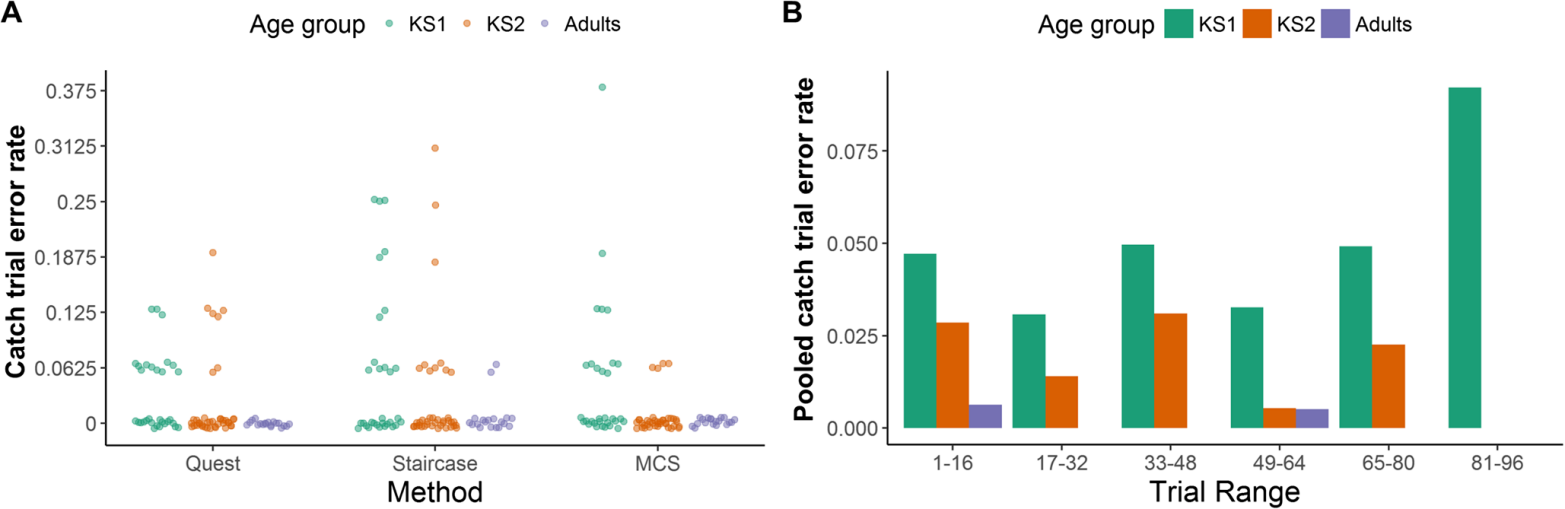
Responses to catch trials. (**A**) The proportion of errors made in 16 catch trials by each participant in KS1 (green; 6–7 years), KS2 (orange; 7–9 years) and adult (purple) groups for each threshold estimation method. Note that values are jittered on the x- and y-axis for display purposes. (**B**) The distribution of errors made across the experimental session. The proportion of errors made in catch trials occurring in each of six bins of trials is shown, pooled across participants and threshold estimation method (i.e., number of errors/total number of catch trials). Note that trials in the final bin (81–96) represent trials in the MCS condition only

To characterize inattentiveness in our child and adult observers, we used the proportion of catch trials for which participants gave an incorrect response for each condition (Fig. 3A; QUEST, staircase, MCS). The errors made in these “easy” catch trials can be used to estimate the lapse rate (proportion of random responding), by multiplying the proportion of errors by two (to account for a 50% guessing rate). Accordingly, the catch trial error rate was significantly correlated with lapse rates estimated during psychometric function fitting, for all methods (QUEST: *r*_s_ = .67, *p* < .001; staircase: *r*_s_ = .70, *p* < .001; MCS: *r*_s_ = .56, *p* < .001). While 52% of participants made no errors in catch trials (i.e., lapse rate = 0), some of the children made multiple errors.

Friedman’s test showed no differences in catch trial errors between the different methods, χ^2^(2) = 1.63, *p* = .44. However, as expected, a Kruskal-Wallis H test showed significant differences in catch trial errors between the age groups, χ^2^(2) = 23.38, *p* < .001. Follow-up comparisons showed that the KS1 children made more errors in catch trials than the KS2 children, Mann-Whitney U = 360.50, *p* = .003 (KS1 mean rank: 43.37; KS2 mean rank: 29.24), and that the KS2 children made more errors than the adults, Mann-Whitney U = 240.00, *p* = .009 (KS2 mean rank: 32.85; adult mean rank: 22.63). These errors occurred throughout the testing session, and were not confined only to earlier or later trials (Fig. 3B). There was some indication that errors increased in the final 81–96 trials, but it is worth noting that these trials were only present in the MCS condition.

### Speed discrimination threshold estimates

**Fig. 4:**
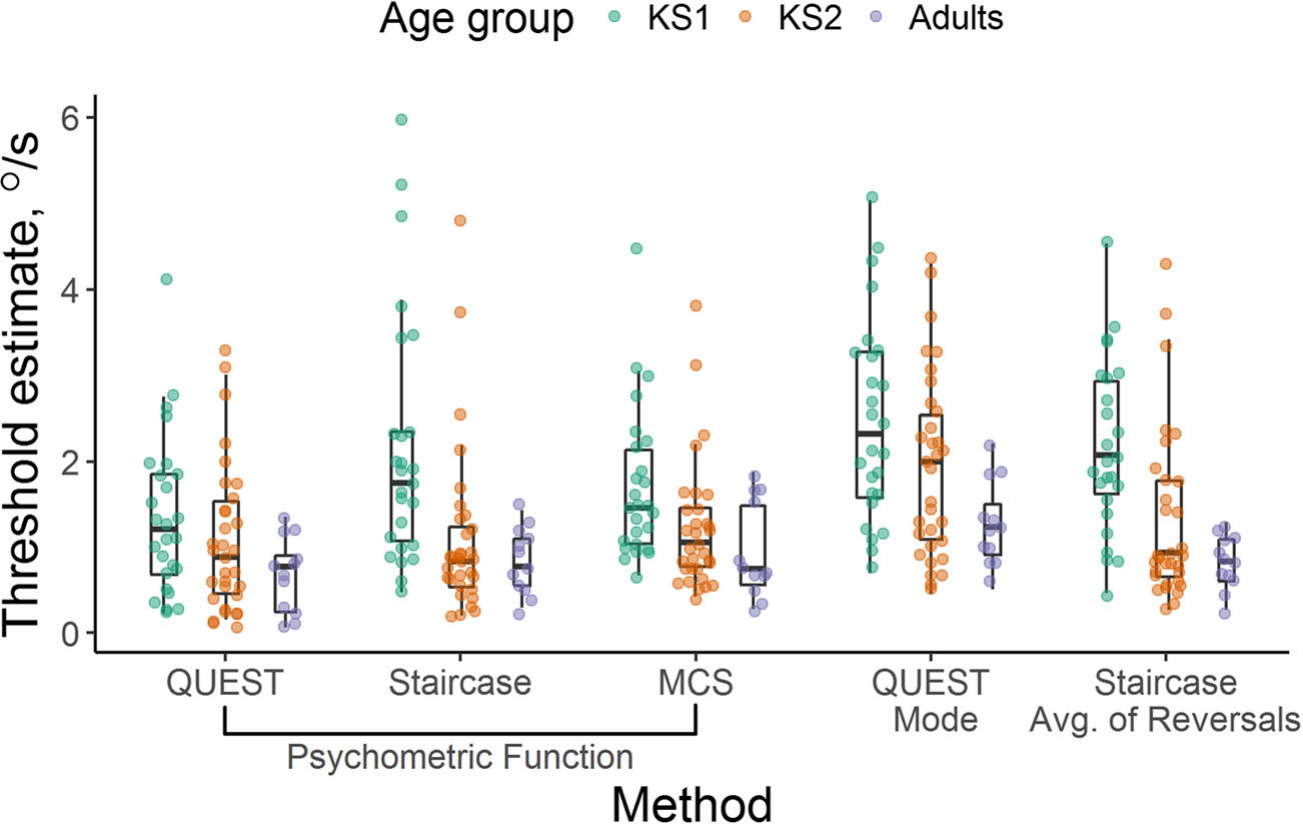
Threshold estimates. Threshold estimates for KS1 (green; 6–7 years), KS2 (orange; 7–9 years) and adult (purple) groups for each threshold estimation method. Box plots show the median, 25th, and 75th percentiles of estimates, and the whiskers extend up to 1.5 times the interquartile range. Box width is proportional to the square-root of the number of points in each group. Note that data are shown here on a linear scale, but were analyzed in log units to minimize the effect of the positive skew

Figure 4 shows individual threshold estimates for each method across age groups. The child groups showed higher inter-subject variability than the adult groups, as expected. To determine whether similar threshold estimates were obtained for the different procedures, and whether the effect of procedure varied across age groups, we conducted a two-way mixed ANOVA on log threshold estimates, with age group as a between-participants factor and procedure as a within-participants factor. First, we looked at threshold estimates obtained when fitting psychometric functions to the datasets obtained from each procedure (Fig. 4). A preliminary analysis showed that there were no age-group differences in the reliability of individual threshold estimates, nor main effects or interactions with method, as indexed by the cumulative probability deviance estimates (*p* > .05). As expected, we found significant age-related improvements in threshold estimates, *F*(2,67) = 9.34, *p* < .001, η_p_^2^ = .22. Planned contrasts showed that the younger, KS1 children (M = 1.57 °/s, 95% CI 1.31–1.85) had significantly higher threshold estimates than the older, KS2 children (M = 1.02 °/s, 95% CI .83–1.23), *p* = .001, but the KS2 children did not differ significantly from adults (M = .77 °/s, 95% CI .50–1.08), *p* = .17. There was also a significant effect of condition, *F*(2,134) = 4.65, *p* = .01, η_p_^2^ = .07, whereby the threshold estimates obtained in the QUEST condition (M = .93 °/s, 95% CI .75–1.12) were lower than those obtained in both the staircase (M = 1.19 °/s, 95% CI .98–1.41, *p* = .01) and MCS (M = 1.20 °/s, 95% CI 1.04–1.37, *p* = .01) conditions. The interaction between age group and condition was not significant, *F*(4,134) = 2.20, *p* = .07, η_p_^2^ = .06. There were significant relationships between the threshold estimates in each condition, using partial correlations to control for age (QUEST and staircase: *r*(67) = .53, *p* < .001; QUEST and MCS: *r*(67) = .36, *p* = .002; staircase and MCS: *r*(67) = .53, *p* < .001). In sum, threshold estimates obtained from post-hoc fitting of psychometric functions reveal age-related changes across all procedures, although lower threshold estimates are obtained in the QUEST condition.

Next, we compared the different ways of estimating thresholds from the QUEST. Unsurprisingly, the threshold estimates obtained from post-hoc fitting of the psychometric function were significantly correlated with the average QUEST mode, while controlling for age, *r*(67) = .67, *p* < .001. A mixed ANOVA with estimation method as a within-participants factor and age group as a between-participants factor revealed differences between the age groups, *F*(2,67) = 5.24, *p* = .008, η_p_^2^ = .14, as before. The younger, KS1 children (M = 1.73 °/s, 95% CI 1.41–2.07) had higher threshold estimates than the older, KS2 children (M = 1.34 °/s, 95% CI 1.09–1.63), although this was not significant, *p* = .07, and the KS2 children had non-significantly higher threshold estimates than the adults (M = .91 °/s, 95% CI .58–1.31, *p* = .07). The source of significant difference between the groups appeared to result from the KS1 children having significantly higher threshold estimates than the adults, *p* = .002. We also found a significant difference between estimation methods, *F*(1,67) = 88.02, *p* < .001, η_p_^2^ = .57, with the QUEST mode leading to higher threshold estimates (M = 1.75 °/s, 95% CI 1.52–2.00) than those derived from the post-hoc fitting of a psychometric function (M = .93 °/s, 95% CI .75–1.12). There was no significant interaction between age group and estimation method, *F*(2,67) = .48, *p* = .62, η_p_^2^ = .01.

Finally, we compared the different ways of estimating thresholds from the staircase (fitting a psychometric function vs. the average of reversals). Note that the average reversals could not be computed for one child (see Data Analysis), so this analysis was conducted on a sample of 25 KS1 children, 32 KS2 children and 12 adults. The two types of threshold estimate were significantly correlated, while controlling for age, *r*(66) = .86, *p* < .001. A mixed ANOVA comparing threshold estimates from the two methods revealed a significant difference in threshold estimates between age groups, *F*(2,66) = 12.44, *p* < .001, η_p_^2^ = .27, with higher threshold estimates in the younger, KS1 children (M = 2.00 °/s, 95% CI 1.63–2.42) than the older, KS2 children (M = 1.07 °/s, 95% CI .83–1.34, *p* < .001), but no significant difference between the KS2 children and the adults (M = .80 °/s, 95% CI .45–1.20, *p* = .23). The staircase average of reversals (M = 1.29 °/s, 95% CI 1.10–1.50) yielded slightly higher threshold estimates than fitting the psychometric function (M = 1.20, 95% CI .99–1.42), but this difference was not significant, *F*(1,66) = 2.45, *p* = .12, η_p_^2^ = .04, and there was no significant interaction between condition and age group, *F*(2,66) = 1.36, *p* = .27, η_p_^2^ = .04. Together, these analyses suggest that the resulting threshold estimates from adaptive techniques led to slightly higher threshold estimates than post-hoc fitting of the psychometric function, although this difference was only significant for the QUEST condition. However, age-related changes were apparent regardless of the threshold estimation technique chosen.

### The relationship between catch trial performance and threshold estimates

To investigate the possible relationship between catch trial performance and threshold estimates, we computed the average catch trial error rate for each participant (across conditions) and conducted non-parametric correlations between this value and threshold estimates while controlling for age. Participants with higher error rates showed significantly higher threshold estimates for all procedures (QUEST psychometric function: *r*_*s*_(67) = .27, *p* = .03; staircase psychometric function: *r*_*s*_(67) = .28, *p* = .02; MCS psychometric function: *r*_*s*_(67) = .36, *p* = .003; staircase average of reversals: *r*_*s*_(66) = .32, *p* = .008) apart from the QUEST average mode, *r*_*s*_(67) = .20, *p* = .10, which did not reach significance. Additionally, to test if catch trial error rates affected our three procedures differently, we divided participants into groups of those who made no errors in any procedure (*n* = 35) and those who made errors in one or more procedure (*n* = 35). We then ran a mixed ANOVA on threshold estimates obtained with psychometric functions with procedure as a within-participants factor, error group as a between-participants factor and age as a covariate. This analysis again showed that threshold estimates were higher with higher error rates, with higher threshold estimates in those who responded incorrectly in one or more catch trials (M = 1.37 °/s, 95% CI 1.15–1.60) than those who did not (M = .97 °/s, 95% CI .79–1.18), *F*(1,67) = 6.55, *p* = .01, η_p_^2^ = .09. However, the effect of error group did not interact with the procedure used, *F*(2,134) = .45, *p* = .64, η_p_^2^ = .01. Further ANOVAs were conducted to confirm that the effect of error group did not vary between the two QUEST threshold estimation techniques and the two staircase threshold estimation techniques (group by procedure interactions: *p* ≥ .57).

### Simulations

Our empirical data suggest that there is a relationship between performance in “easy” catch trials and threshold estimates. However, it is difficult to fully equate the proportion of errors in catch trials to the lapse rate, as high error rates could also reflect other factors, such as extreme difficulty with the task (although note that all participants passed a criterion of consecutive responses in the practice phase), forgetting the response strategy, or generally reduced motivation across the board. Moreover, the number of catch trials had to be kept low to avoid overly long testing time, so it is possible that children responding correctly to catch trials made lapses at other points throughout the session. Simulations allowed us to more clearly investigate the effects of attentional lapses, by assessing the effect of random responses on occasional trials, with a known threshold that was held constant. Therefore, we could address whether increased lapse rates could explain the age-related differences in threshold estimates obtained in our participants.

**Fig. 5:**
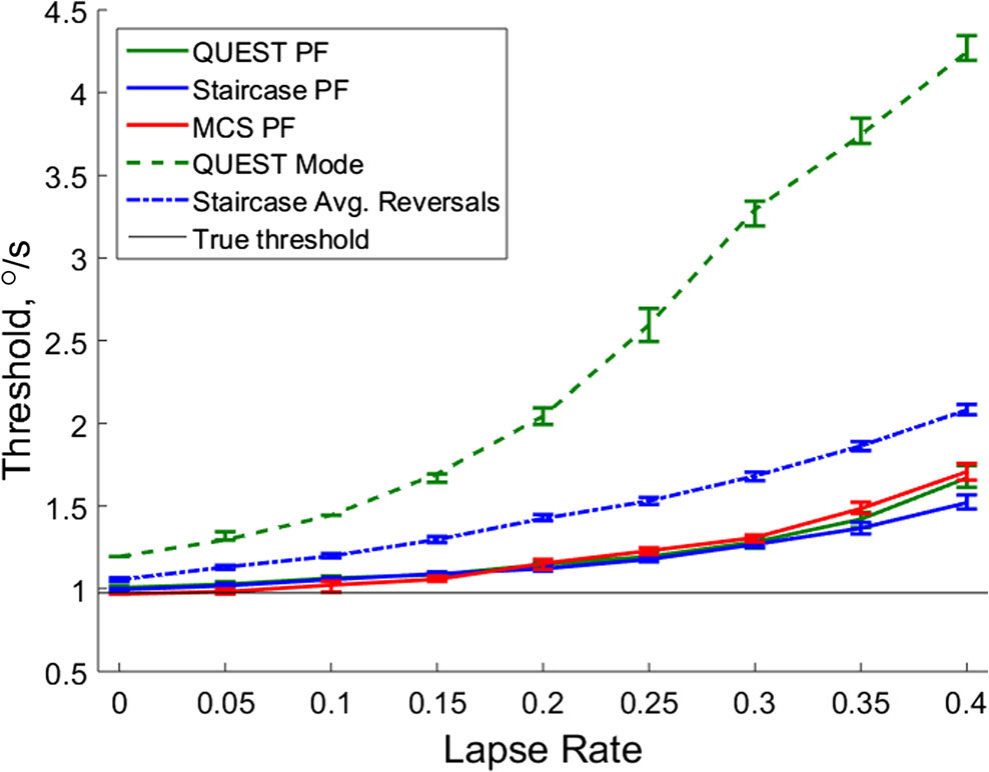
The effect of increasing lapse rates on a simulated observer. The effect of increasing lapse rates on the threshold estimates of a simulated observer with a true threshold of .97 °/s. 2,000 simulations were run for each lapse rate and for each of the three procedures (QUEST, staircase, MCS). Thresholds were estimated with identical parameters and exclusion criteria as the experimental data. For each lapse rate, the median threshold estimate is plotted with 95% bootstrapped confidence intervals around the median. *PF* psychometric function

These simulations assessed the effects of differing lapse rates on threshold estimates on a simulated observer, using the same task parameters and threshold estimation procedures as for the youngest, KS1 children (Fig. 5). Table 1 shows that relatively more simulated datasets were excluded from the staircase method than the other techniques, which was also apparent in our empirical datasets. As expected, threshold estimates increased with increasing lapse rates for all methods, becoming further from the true threshold, and became more variable. These simulations therefore demonstrate that increased lapses can cause increased threshold estimates. First, we considered whether the effect of increasing lapse rates differentially affected the various methods, potentially explaining the different threshold estimates obtained in our experimental data (Fig. 4). While our main analysis may have lacked power to detect such differences due to low lapse rates, simulations do not suffer from this drawback.

**Table 1:** Percentage (%) of simulated datasets excluded for different levels of lapse rate

	Lapse rate								
Method	0	0.05	0.1	0.15	0.2	0.25	0.3	0.35	0.4
QUEST PF	0	0	0	0.2	0.8	1.3	2.4	4.9	8.6
Staircase PF	5.6	3.9	4.6	4.0	5.6	7.7	10.4	13.6	16.9
MCS PF	0	0	0.1	0.1	0.6	1.2	1.9	3.1	5.9
QUEST mode	0	0	0	0	0.2	0.5	0.7	1.9	3.9
Staircase Avg. Reversals	2.2	1.8	2.2	2.7	2.0	2.7	1.7	1.8	1.3

*Note.* Simulations were excluded if the threshold estimate fell outside of the stimulus range and/or if negative psychometric function slopes were obtained, or if too few staircase reversals were obtained in the staircase average of reversals condition

In our experimental data, we found that lower threshold estimates were obtained from psychometric functions fit to the data obtained with the QUEST, compared to the staircase and MCS procedures. In the simulations, there was no evidence for this effect. Instead, the MCS threshold estimates were lower than staircase and QUEST for low levels of lapse rate (0–0.15) and the staircase threshold estimates were lowest at higher levels of lapse (0.2–0.4). Generally, the difference between procedures became more pronounced at higher levels of lapse rate. For example, the QUEST and staircase estimates were separated by only 0.01 °/s at a lapse rate of 0, but by 0.15 °/s at a lapse rate of 0.4, and the staircase and MCS methods were separated by 0.03 °/s at a lapse rate of 0 and 0.19 °/s at a lapse rate of 0.4.

As in our experimental data, the thresholds estimated using the mode of the QUEST posterior distribution were considerably higher than the thresholds estimated with post-hoc fitted psychometric functions, and this was true for all levels of lapse rate. Follow-up simulations showed that the difference was largely due to the slope of the Weibull function (beta value) assumed by the QUEST function (Supplemental Material). However, these simulations also showed that, regardless of the slope value used, QUEST mode was considerably more affected by increasing lapse rates than the same data refitted with a psychometric function (Supplemental Material). The staircase average of reversals also led to slightly higher threshold estimates than those obtained from the psychometric functions, which was a non-significant trend in the experimental data, and could potentially be attributed to too few staircase reversals (Witton et al., 2017) and/or a suboptimal ratio of stepsize to slope (spread) of the observer’s psychometric function (García-Pérez, 2011). For the QUEST procedure, the QUEST mode estimate was 0.19 °/s higher than the threshold estimated from the psychometric function at a lapse rate of 0, and this steadily increased to a difference of 2.57 °/s for a lapse rate of 0.4. The discrepancy in staircase estimates was relatively less affected by lapse rate, but increased steadily from 0.06 °/s at a lapse rate of 0 to 0.56 °/s at the highest lapse rate tested (0.4). In sum, our simulation results suggest that increased attentional lapses exaggerate discrepancies in threshold estimates. The QUEST mode appears to be particularly affected by increased lapse rates, with the largest increases in bias.

We considered two possibilities that might have accounted for the reduced bias in threshold estimates obtained from fitting a psychometric function compared to the QUEST mode or staircase average reversals. First, it is possible that post-hoc fitting of the psychometric function led to less biased threshold estimates with increasing lapses because lapse rate was a free parameter (allowed to vary between 0 and 0.1) in the fitting of the psychometric function. However, even when the psychometric functions were fit with the lapse rate fixed at zero, threshold estimates were less biased than the staircase average of reversals and QUEST mode (see Supplemental Material). Second, it could be that the reduction of bias associated with post-hoc fitting of a psychometric function is contingent on the inclusion of catch trials in the fitting procedure. However, additional simulations showed that the thresholds estimated from post-hoc fitting of the psychometric function were less biased than the QUEST mode and average of staircase reversals even when the catch trials were removed and replaced with extra trials in the adaptive tracks (see Supplemental Material). Therefore, the benefit of fitting psychometric functions is not dependent on either of these factors.

As shown in Fig. 3, most participants made no errors in the 16 catch trials, and those who did make errors generally made only one or two errors (corresponding to a lapse rate of 12.5% and 25%, respectively). Figure 5 shows that even low lapse rates can bias threshold estimates, but the magnitude of this bias is relatively small, particularly when fitting psychometric functions. Specifically, the increase in threshold estimates obtained from psychometric functions between a lapse rate of 0 and a lapse rate of 0.25 was .19 °/s, .18 °/s, and .26 °/s for the QUEST, staircase, and MCS, respectively. In contrast, age-related differences in speed discrimination threshold estimates far exceed this value, with adults having thresholds on average 0.8 °/s lower than KS1 children. Thus, the effect of lapsing on 25% of trials is about a quarter of the developmental age effect. We therefore reason that developmental changes in speed discrimination threshold estimates are unlikely to be fully attributable to differences in attentiveness, and are likely to reflect, at least in part, real changes in perceptual abilities with age.

## Discussion

In this study, we estimated speed discrimination thresholds in children (KS1: 6–7 years; KS2: 7–9 years) and adults, using QUEST, staircase, and MCS procedures. We used the proportion of errors on catch trials as a coarse measure of inattentiveness. As expected, both child groups made more catch trial errors compared to adults, and these errors occurred throughout the test session. Individuals with higher catch trial error rates generally had higher threshold estimates. Lower threshold estimates (higher sensitivities) were obtained, overall, from psychometric functions in the QUEST condition than the staircase and MCS conditions. Additionally, lower threshold estimates were obtained when refitting a psychometric function to the QUEST data than when using the QUEST mode. Importantly, however, developmental improvements in speed discrimination threshold estimates were apparent across all psychophysical procedures used, with no significant interactions between age group and procedure. We also investigated whether differences between threshold estimation techniques could be explained by differential effects of attentional lapses. We found that threshold estimates were particularly elevated away from the true threshold as a function of increasing lapses when using the QUEST mode or average of staircase reversals, compared to thresholds estimated when fitting a psychometric function to the data, post-hoc.

The fact that some threshold estimation techniques are more affected by lapses than others could partly explain the discrepant threshold estimates obtained in our experimental data. For example, attentional lapses could contribute to particularly elevated threshold estimates obtained from the QUEST mode, while thresholds estimated based on fitting psychometric functions to the same data remain closer to the true threshold. However, in this case, lapses cannot fully explain the observed differences between QUEST threshold estimates, because our simulations showed differences even when the simulated observer had a zero lapse rate, which appeared to arise due to the choice of slope (beta) parameter used in the QUEST function. Nonetheless, nonzero lapse rates exacerbate differences between the threshold estimation techniques. Notably, we did not observe any significant interactions between threshold estimation technique and age group in our experimental data, which we would have expected given that (a) children had more frequent catch trial errors than adults, suggesting increased attentional lapses, and (b) lapses have differential effects on threshold estimation techniques. The absence of significant interaction effects could be a result of low power, especially if most children made few attentional lapses. Additionally, the finding that psychometric functions yielded lower threshold estimates in the QUEST condition than the staircase and MCS conditions in the experimental data was not borne out in the simulated datasets at any level of lapse rate, suggesting that the patterns observed in our behavioral data could not be explained by a differential effect of attentiveness on these three methods.

Our finding that thresholds estimated after fitting psychometric functions are more immune to attentional lapses than threshold estimates obtained at the end of adaptive tracks (QUEST mode, average of staircase reversals) supports Dakin and Frith’s (2005) recommendation to fit the whole psychometric function when testing populations for whom attentional lapses might be elevated. Fitting the psychometric function allows the lapse rate to be modelled, as we did here by setting the lapse rate to be a free parameter between 0 and 0.10. However, even when this lapse rate parameter was fixed, fitting psychometric functions led to less biased threshold estimates than the QUEST mode or staircase average of reversals – perhaps because it allows for differences in psychometric function slope. Rather than solely recommending MCS for fitting psychometric functions (cf. Dakin & Frith, 2005), we show that the same principle can be achieved by fitting a psychometric function to the data from adaptive methods, as suggested by Hall (1981; Amitay et al., 2006). This approach has a clear benefit that the researcher does not have to commit to a set of testing values in advance – which might be particularly difficult if the researcher does not have previous studies to know what range of values to expect, and/or if there is much between-participants variability.

The difference in threshold estimates for different psychophysical procedures found here contrasts with Buss et al. (2001), who showed that comparable threshold estimates were obtained across three procedures for children aged 6–11 years and adults. However, there are numerous differences between Buss et al.’s study and the current study, four of which we consider here. First, Buss et al. compared different procedures to those compared here (a 1-up 3-down staircase, a MLE procedure and MCS), and while Buss et al. used between three and four adaptive tracks, we used only two. Second, Buss et al. used an auditory detection task with feedback, as opposed to the visual discrimination task without feedback employed here. Third, Buss et al. used a three-alternative-forced choice procedure as opposed to our two-alternative-forced-choice procedure, thus tracking a higher level of performance. Finally, we saw a larger sample of children with narrower age groups. More research is needed to investigate the importance of each of these differences and how they interact. It has been suggested that better threshold estimates are achieved with three or more alternatives, compared to two alternatives, both in adults (Jäkel & Wichmann, 2006; Kollmeier et al., 1988; Leek et al., 1992; Leek, 2001; Shelton & Scarrow, 1984) and children (Buss et al., 2012; Sutcliffe & Bishop, 2005), which could explain the discrepancy in results. However, two-alternative and three-alternative methods have not been compared directly in the visual domain in children.

It remains unclear why we obtained lower threshold estimates from psychometric functions in the QUEST condition compared to the staircase and MCS procedures in our observers, but not in the simulated datasets. We have established that it cannot be due to differential effects of attentional lapses, as the staircase yielded the lowest threshold estimates at high levels of lapse rate in the simulated datasets. However, there could be additional factors that affect a real observer, which are not taken into account by the simulations. Indeed, there are previous reports of lower threshold estimates in adaptive procedures than MCS procedures, even when simulation studies suggest that similar threshold estimates should be obtained (Kollmeier et al., 1988; Stillman, 1989; Taylor et al., 1983; see also Leek, 2001, for review). In QUEST, the presentation of trials is dependent on performance, whereas this is not the case for MCS, where there may be too many easy and/or difficult trials for a given observer (depending on the levels of signal strength chosen), potentially leading to reduced motivation. However, it is less apparent why QUEST might lead to improved performance compared to the staircase, which is also an adaptive procedure. Perhaps the use of jitter in the QUEST meant that a greater range of trial intensities were presented compared to that in the staircase method, reducing boredom and/or providing reminders of the cue. Other factors, such as nonstationary thresholds (Fründ et al., 2011; Hall, 1983; Leek et al., 1991; Taylor et al., 1983; Wallis et al., 2013) and response biases (e.g., a preference to respond “faster” for a certain stimulus location or interval, or to alternate responses [Tune, 1964; Raviv et al., 2014]; serial inter-trial dependencies (Baird, Green, & Luce, 1980; Fründ, Wichmann, & Macke, 2014; Fischer & Whitney, 2014; Green, Luce, & Duncan, 1977; Jones, Moore, Shub, & Amitay, 2015a; Stewart, Brown, & Chater, 2002) could also differentially impact the various psychophysical procedures, although this was beyond the scope of the current research. Additional factors such as the starting point for adaptive tracks (Leek, Hanna, & Marshall, 1992) may also affect the performance of an observer in a way that was not captured by our simulations.

Although inappropriate choices of QUEST slope (beta) values can lead to biased threshold estimates, our data show that estimating thresholds through post-hoc psychometric function fitting minimizes this effect. This is important because children vary from adults in the slope of their psychometric functions (Buss et al., 2009), and it may be difficult to select a single QUEST slope value that is appropriate for all observers. Similarly, bias in the average of staircase reversals, which has been shown to be introduced by an inappropriate ratio of stepsize to observer’s slope (García-Pérez, 2011), can be minimized significantly by fitting the same data with a psychometric function. These findings are in line with the overall conclusion that fitting psychometric functions of efficient adaptive methods is a powerful approach that yields highly robust results – provided that lapse rates are within a reasonable range (< 20%). Although rarely used with child populations, adaptive procedures that estimate both the threshold and slope of the psychometric function (Brand & Kollmeier, 2002; King-Smith & Rose, 1997; Kontsevich & Tyler, 1999; Shen & Richards, 2012) may also confer benefits for child observers with variable slopes. However, it will be necessary to weigh such benefits against the cost of increased test durations which are likely to be detrimental in populations with limited attention (Jones, Kalwarowsky, Braddick, Atkinson, & Nardini, 2015b).

Overall, we have shown that attentional lapses are more common in children than adults and that this leads to biased threshold estimates, especially for methods that do not fit a psychometric function to the data. Additionally, differences between methods become more pronounced when lapse rates are higher. Particularly high lapse rates (and therefore more inaccurate threshold estimates) could be obtained in studies which use tasks that are less engaging and motivating for child observers, and in studies of children with developmental conditions, for whom attentional difficulties have been reported (for reviews, see Amso & Scerif, 2015; Cornish et al., 2012). Indeed, future research is needed to consider the effects of inattention on threshold estimates in atypically developing groups, to ensure that reports of reduced sensitivity compared to typically developing children do not arise purely due to a higher proportion of attentional lapses (see Roach et al., 2004; Skottun & Skoyles, 2007; Sutcliffe et al., 2006).

While we used speed discrimination in this study as an example task to assess the influence of attentional lapses on perceptual performance across methods, we can also gain insights to further our understanding of the development of children’s speed discrimination abilities. We demonstrated that attentional lapses can cause thresholds to be underestimated, as shown previously (e.g., Kingdom & Prins, 2010; Madigan & Williams, 1987), and our experimental catch trial data suggest that attentional lapses are higher in young children. However, most children made no errors in catch trials, suggesting low lapse rates. Low lapse rates lead to biases that are considerably smaller than the age-related changes in threshold estimates reported. In addition, developmental changes in speed discrimination thresholds were evident regardless of the psychophysical procedure chosen. Therefore, it appears that developmental changes in speed discrimination threshold estimates cannot be fully attributable to differences in attentiveness between children and adults, or differences in procedures, and thus likely reflect, at least in part, real age-related changes in perceptual abilities.

On the basis of our experimental and simulated data, we recommend fitting a psychometric function to estimate thresholds, especially if it is suspected that lapses might be elevated, as in children or clinical groups. We have shown that this is a feasible strategy even for data collected with adaptive procedures like QUEST and staircases. The inclusion of catch trials provides a coarse but useful measure of attentiveness, although the benefits of fitting psychometric functions can be obtained even without their inclusion.

## Footnotes

1 Key Stages refer to the division of the UK National Curriculum, with KS1 and KS2 corresponding to 5–7 years and 7–11 years, respectively.

2 The QUEST mean (King-Smith et al. 1994) is not reported here because it often fell outside of the stimulus range.
